# Hawley’s Vaginal Syringe

**Published:** 1886-05

**Authors:** 


### Hawley's Vaginal Syringe.

We have examined, with the care arising from a deep interest in the special work which this syringe aims to accomplish, the vaginal syringe to which J. A. Hawley & Co., of Canandaigua, N. Y., direct the attention of the profession. To secure a perfect vaginal douche, without the cumbersome accessories of rubber cloth, receivers, etc., which are necessary for the protection of the patient, is a desideratum long aimed at, and heretofore not attained. This syringe, in the simplicity of its arrangement and its compactness and cheapness, comes nearer the ideal than anything we have examined. It consists of a rubber cylindrical speculum, small in size, in order to secure ease of introduction, to which is attached two rubber bulbs, by the compression and expansion of which water is sucked up and forced into the vagina. The quantity of water required is small, while, in its use, there is avoided the wetting of the patient's linen, thorough cleansing of the vaginal surface being efficiently accomplished. We think favorably of the plan and the instrument, and thus far in our practice it has fulfilled the purposes designed by the proprietor.

